# Editorial: Immune-gut-brain axis - a key player in overall human pathologies

**DOI:** 10.3389/fimmu.2026.1879531

**Published:** 2026-07-09

**Authors:** A. Raquel Esteves, Vitor Cabral, Mario F. Munoz Pinto, Ana Raquel Santiago

**Affiliations:** 1 Centre for Neuroscience and Cell Biology, University of Coimbra, Coimbra, Portugal; 2 Universidade de Coimbra Centro de Inovacao em Biomedicina e Biotecnologia, Coimbra, Portugal; 3 Siec Badawcza Lukasiewicz - PORT Polski Osrodek Rozwoju Technologii, Wrocław, Poland; 4 Departamento de Bioquímica y Biología Molecular, Facultad de Farmacia, Universidad de Sevilla, and Instituto de Biomedicina de Sevilla-Hospital Universitario Virgen del Rocío/CSIC/Universidad de Sevilla, Sevilla, Spain; 5 Universidade de Coimbra Faculdade de Medicina, Coimbra, Portugal

**Keywords:** gut microbiota, dysbiosis-related diseases, gut-brain axis, microbiota-immune modulation, neuroimmune interactions, neuroinflammation

The study of the immune–gut–brain axis has rapidly evolved into a unifying framework that is reshaping our understanding of human disease. We now know that this bidirectional network links microbial ecosystems, immune signaling, metabolic pathways, and neural function, and it is not only confined to gastrointestinal and neurological disorders but has emerged as a key determinant of health and disease across the lifespan. In this Research Topic, “Immune–Gut–Brain Axis: A Key Player in Overall Human Pathologies,” several contributions collectively underscore the breadth, complexity, and translational relevance of this field. The studies assembled here comprise 6 original research papers and 5 reviews, covering a wide range of topics, from descriptive microbiome research to mechanistic and clinical insights across different human disorders.

Neurodevelopmental disorders and early-life programming as a critical window for immune–gut–brain interactions represent a major focus of this Research Topic. Liu et al. provide a systematic review on autism spectrum disorder (ASD) and gut microbiota and present a deep and global perspective on how gut microbiota alterations and the gut–brain axis are linked to ASD-relevant phenotypes. Moreover, it identifies major contributors such as fecal microbiota transplantation, maternal immune activation, and metabolic comorbidities, highlighting how neurodevelopmental disorders are systemic and multi-organ. Complementing this, this Research Topic also includes research that combines metagenomic and untargeted metabolomic analyses, revealing gut microbiota dysbiosis, functional impairment, and metabolic disturbance in pediatric neurodevelopmental disorders (NDDs). In this work, Wang et al. also provide a foundation for microbiota- and metabolite-targeted therapeutic strategies in childhood NDDs. Extending this developmental perspective, a study on gestational diabetes mellitus (GDM) and its impact on maternal and offspring immunity points to increased small intestinal permeability, altered immune responses, and gut microbiome alterations in children of mothers with GDM. Vorobjova et al. suggest that maternal metabolic status may imprint long-term effects on gut barrier function and immune development, reinforcing the notion that the immune–gut–brain axis is shaped early in life.

The importance of immune-mediated communication along the gut–brain axis is further emphasized in studies addressing neurodegenerative and neuroinflammatory conditions. Using a colitis-induced model of Parkinson’s disease (PD), induced by peripheral inflammation triggered by dextran sodium sulphate administration, Espinosa-Oliva et al. show how sex influences peripheral inflammation and affects PD progression and neuropathology. These findings challenge simplified interpretations of disease progression and reinforce the need to integrate biological variables such as sex into both experimental design and therapeutic strategies. Similarly, using a cuprizone-induced multiple sclerosis (MS) model, Ferreira et al. highlight the relevance of the temporal coordination between gut and brain pathology. Furthermore, it demonstrates that gut barrier disruption, microbial alterations, mucosal immunity, inflammation, and changes in oxidative stress-related enzymes parallel both demyelination and remyelination processes, gliosis, and related molecular changes, suggesting that the gut is not merely a trigger but an active participant throughout disease evolution. In addition, Ren et al. discuss and review how dysfunction in bidirectional communication between the gut microbiome and the central nervous system, particularly involving the blood–brain and gut barriers, contributes to MS pathophysiology.

Beyond neurological disorders, this Research Topic expands the relevance of the immune–gut–brain axis to systemic, cardiometabolic, infectious, and stress-related conditions. In the case–control study in elderly hypertensive patients, Cheng et al. illustrate how gut microbiota dysbiosis and systemic inflammation are intertwined as key pathophysiological features. Moreover, this research article provides groundwork for identifying microbial signatures that will help develop microbiota-based diagnostic and more effective, accessible, and personalized therapeutic strategies. Herein, it is also discussed how this axis plays a crucial role in infectious and stress-related conditions. In the context of infectious disease, the review on cryptococcal meningitis, a fatal central nervous system infection, systematically integrates immune responses, metabolic reprogramming, and microbiota dynamics into a comprehensive model of host–pathogen microenvironment interaction and regulation. Wen et al. propose a combined therapeutic strategy of immunomodulators, metabolic inhibitors, and microbiota intervention that moves beyond conventional approaches. In parallel, the mini-review on stress-induced ulcer damage includes clinical and preclinical studies related to stress ulcers and the gut–brain axis. Moreover, Abdel-Sater and Hassan highlight how microbial-derived neurotransmitters and metabolites mediate the impact of stress responses on mucosal integrity, neuroendocrine signaling, and gut health.

Another recurring theme of this Research Topic is the therapeutic modulation of the microbiome. A pilot study examining autologous hematopoietic stem cell transplantation (AHSCT) in MS patients has been shown to arrest inflammation and induce long-term therapeutic benefit. However, how this affects the gut microbiome has never been explored. Hence, Yan et al. investigate microbiome dynamics in MS patients undergoing AHSCT and provide preliminary evidence that immune-resetting therapies may also reshape microbial ecosystems. In addition, this study identifies specific microbiome changes that reflect treatment status or disease activity in MS. Alongside this, the review article on Alzheimer’s disease points toward a future in which targeting the gut microbiota becomes an integral component of precision medicine. Herein, Lei et al. highlight emerging microbiome-based interventions such as probiotics and fecal microbiota transplantation, demonstrating neuroprotective effects by reducing neuroinflammation and restoring cognitive function in preclinical studies. It also reinforces the need for randomized controlled trials and addresses challenges and future directions.

Despite these advances, several challenges remain. A lack of standardization in microbiome methodologies, limited longitudinal data, and the need for integrative multi-omics approaches continue to hinder translation. Moreover, establishing mechanistic causality, rather than association, remains a critical hurdle. Addressing these gaps will require larger cohort studies and thoroughly designed clinical trials. Overall, the articles in this Research Topic collectively reinforce the immune–gut–brain axis as an understudied central hub in human pathophysiology. By integrating insights from neurodevelopmental, neurodegenerative, metabolic, infectious, and stress-related disorders, they highlight both the universality and specificity of this axis across diseases. As the field advances, a systems-level understanding that incorporates microbial ecology, immune regulation, and neural function will be essential for developing innovative prognostic or diagnostic tools and therapeutic strategies. The immune–gut–brain axis is no longer an emerging concept; it is a cornerstone of modern biomedical research, poised to redefine the future of medicine.

